# Delayed-release budesonide combined with cyclophosphamide in a patient with IgA nephropathy with eGFR < 30 mL/min/1.73 m²: A case report

**DOI:** 10.1097/MD.0000000000047060

**Published:** 2026-01-16

**Authors:** Zhuan'e Yao, Pengbo Wang, Qinjuan Fu, Peng Zhang

**Affiliations:** a Department of Nephrology, Shaanxi Provincial People’s Hospital, Xi’an, Shaanxi, China; b Department of Nephrology, Shaanxi Second Provincial People’s Hospital, Xi’an, Shaanxi, China.

**Keywords:** case report, cyclophosphamide, delayed-release budesonide, IgA nephropathy

## Abstract

**Rationale:**

Delayed-release budesonide, as a novel oral agent, may be an effective treatment for IgA nephropathy. Its efficacy and safety in adult IgA nephropathy patients with estimated glomerular filtration rate (eGFR) > 35 mL/min/1.73 m² has been previously confirmed in the phase III clinical study (NeflgArd). For patients at high risk of progression to end-stage renal disease, it is uncertain whether delayed-release budesonide combined with cyclophosphamide on the basis of supportive therapy could improve the prognosis of adult IgA nephropathy patients.

**Patient concerns:**

A 37-year-old male presented with fever (max 38.2°C) and sore throat, urinalysis revealed abnormalities.

**Diagnoses:**

Laboratory tests showed urinary protein of 2.98 g/d, serum creatinine of 321 μmol/L, eGFR of 20.27 mL/min/1.73 m^2^, plasma albumin 34.6 g/L. Renal biopsy pathology showed sclerosing IgA nephropathy (Lee grade Ⅳ, Oxford classification M0E0S1T1C0).

**Interventions:**

The patient was treated with delayed-release budesonide (16 mg/d) for 36 weeks, in combination with intravenous cyclophosphamide (0.8 g monthly for 6 months).

**Outcomes:**

After 36 weeks of therapy, the eGFR increased from 20.27 to 48.12 mL/min/1.73 m^2^, serum creatinine decreased from 321 to 157 μmol/L, urinary protein decreased from 2.98 to 0.378 g/d, and plasma albumin increased from 34.6 to 45.6 g/L. During the course of treatment, no significant drug-related adverse reactions were observed, except for a mild cold.

**Lessons:**

This case suggests that delayed-release budesonide in combination with cyclophosphamide on the basis of supportive therapy is effective in treating adult IgA nephropathy patient with eGFR < 30 mL/min/1.73 m^2^. This approach warrants further investigation in larger prospective studies.

## 1. Background

IgA nephropathy, as an immune-mediated renal disease, is the most common primary glomerular disease, with the main clinical manifestations of hematuria or proteinuria, and with the aggravation of the disease can develop into renal failure.^[1]^ The 2021 Kidney Disease: Improving Global Outcomes (KDIGO) guidelines recommend that patients with IgA nephropathy with urinary protein > 0.5g/d, should be treated with angiotensin-converting enzyme inhibitors/angiotensin II receptor blockers and sodium glucose cotransporter-2 inhibitor, and glucocorticoids should be considered for patients at a high risk of renal progression. However, the long-term efficacy and safety of glucocorticoids remains controversial, especially for patients with estimated glomerular filtration rate (eGFR) < 30 mL/min/1.73 m², the application should be more cautious.^[2]^ Patients with IgA nephropathy who have more urinary protein and poor initial renal function are at a high risk of progressing to end-stage renal disease.^[3]^ Previous studies have shown that glucocorticoids combined with cyclophosphamide can be effective in treating patients with IgA nephropathy accompanied by abnormal renal function.^[4,5]^ However, the combination therapy also has side effects such as infection, leukopenia, and malignant tumor. For IgA nephropathy patients with massive proteinuria and severely impaired renal function, simply giving supportive therapy has poor efficacy, for patients like us with an eGFR < 30 mL/min/1.73 m² and high proteinuria, the risk of progression remains substantial even in the absence of crescent formation, the optimal initial immunosuppressive therapy remains controversial, and the use of high-dose systemic corticosteroids is considered to carry an unacceptable risk of adverse events relative to their uncertain benefits. Although delayed-release budesonide offers a safer alternative choice, its efficacy as monotherapy in such advanced stages of kidney disease remains unclear. The study by Jia Q and Ma F et al indicates that for patients with IgA nephropathy in stages 3 to 4 chronic kidney disease, glucocorticoid combined with cyclophosphamide can effectively stabilize or improve renal function.^[4,5]^ Based on the existing literatures and the high-risk status of our patient, we considered a treatment approach combining delayed-release budesonide with systemic immunosuppression.

Delayed-release budesonide is an enteric mucosal B-lymphocyte immunomodulator, which can reduce the production of pathogenic galactose-deficient IgA1 (Gd-IgA1) by inhibiting the number and activity of B-lymphocytes, and treat IgA nephropathy from the source.^[6,7]^ In 2021, delayed-release budesonide received expedited regulatory approval for the treatment of proteinuria in patients with IgA nephropathy and an eGFR > 30 mL/min/1.73 m²,^[8]^ however its efficacy and safety in patients with eGFR < 30 mL/min/1.73 m² has been less well reported. This report describes the case of a 37-year-old man with IgA nephropathy, proteinuria, and an eGFR of 20.27 mL/min/1.73 m^2^ treated with delayed-release budesonide combined with cyclophosphamide.

## 2. Case report

**Figure 1.**
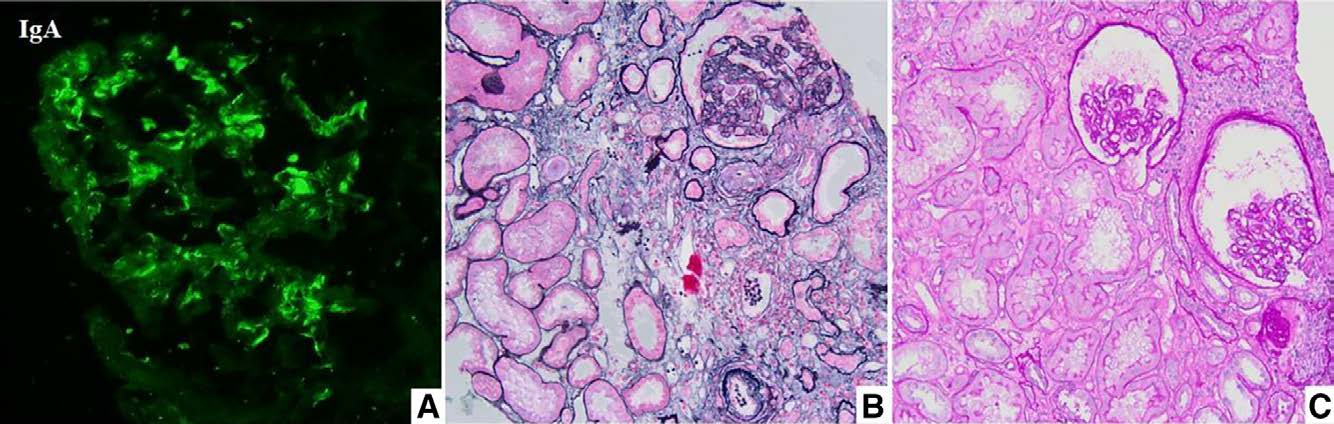
The pathological findings of renal biopsy. (A) Immunofluorescence staining showed that the IgA is positive along the mesangial and subendothelial of glomeruli (×400). (B) Light microscopy showed that segmental glomerulosclerosis with adjacent podocyte hyperplasia, multifocal tubular atrophy with interstitial fibrosis, and arteriolar hyalinization (PASM + Masson, ×200). (C) The glomerular capillary loops showed ischemic sclerosis and wrinkling (PAS, ×200). PAS = periodic acid-Schiff, PASM = periodic acid-silver methenamine,

A 37-year-old Chinese male complained of fever and abnormal urinalysis for 5 days, with a maximum temperature of 38.2°C, accompanied by sore throat, hypertension, and a Body Mass Index of 28.6 kg/m^2^. We examined urinary erythrocytes 2+, urinary proteins 2+, urinary proteins 2.98 g/d, serum creatinine 321 µmol/L, plasma albumin 34.6 g/L and eGFR 20.27 mL/min/1.73 m^2^, anti-neutrophil cytoplasmic antibodies, anti-glomerular basement membrane antibodies, complement, and autoantibody series were normal, hepatitis B virus and hepatitis C virus were negative. Renal biopsy was performed, and pathological results indicated sclerosing IgA nephropathy (Lee grade Ⅳ, Oxford classification M0E0S1T1C0; Fig. 1). The main supportive treatment is Irbesartan 150 mg/d, and after upper respiratory tract infection was cured, delayed-release budesonide (16 mg/d), oral treatment for 36 weeks, while monthly intravenous injection of cyclophosphamide (0.8 g/mo), for a total of 6 months (cumulative dose 4.8 g). Before starting the treatment of cyclophosphamide, we discussed the potential risk of drug-induced infertility with the patient. The patient stated that his family planning goals had been achieved, expressed no concern regarding cyclophosphamide-related fertility risks, and provided informed consent for treatment. All drug dosages remained unchanged throughout the treatment and observation periods.

**Figure 2.**
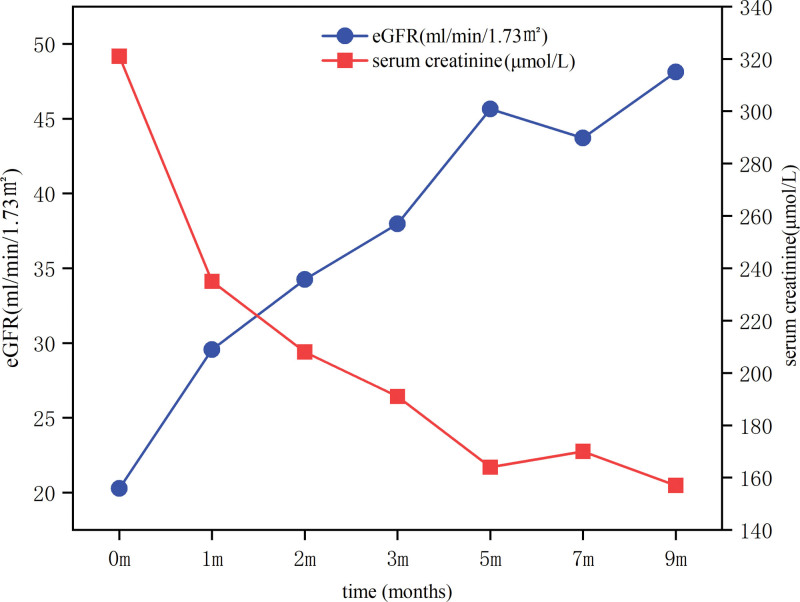
Longitudinal trends in eGFR (estimated glomerular filtration rate, mL/min/1.73 m²) and serum creatinine levels (μmol/L) before and after the treatment of delayed-release budesonide combined with cyclophosphamide. The blue line represents eGFR and the red line represents serum creatinine levels, which shows the changes from December 2023 to September 2024.

**Figure 3.**
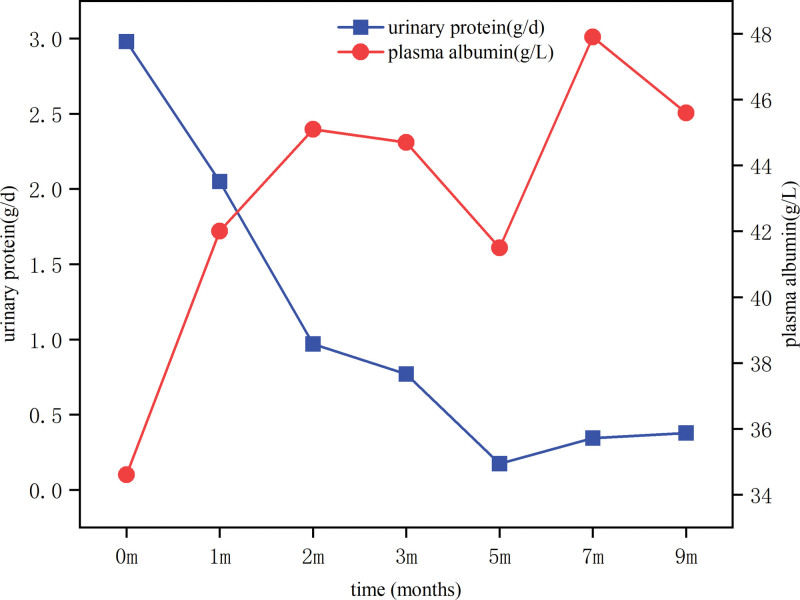
Longitudinal trends in urinary protein (g/d) and plasma albumin levels (g/L) before and after the treatment of delayed-release budesonide combined with cyclophosphamide. The blue line represents urinary protein and the red line represents plasma albumin levels, which shows the changes from December 2023 to September 2024.

After 36-weeks of early treatment with delayed-release budesonide combined with cyclophosphamide on the basis of supportive treatment, the patient’s eGFR level rose from 20.27 mL/min/1.73 m^2^ at diagnosis to 48.12 mL/min/1.73 m^2^, the serum creatinine level decreased from the starting level of 321 to 157 µmol/L, urinary protein decreased from 2.98 to 0.378 g/d, and plasma albumin increased from 34.6 to 45.6 g/L, and the longitudinal changes in laboratory parameters throughout the treatment course are summarized in Table 1, the longitudinal trends in eGFR and serum creatinine levels are shown in Figure 2, the urinary protein and plasma albumin levels are shown in Figure 3. It is worth mentioning that no significant drug-related adverse reactions were observed during the 36-weeks follow-up except for a mild cold.

**Table 1:** Longitudinal changes in laboratory parameters during treatment with delayed-release budesonide and cyclophosphamide.

Parameter	Baseline (mo 0)	mo 1	mo 2	mo 3	mo 5	mo 7	mo 9
eGFR (mL/min/1.73 m²)	20.27	29.55	34.25	37.97	45.65	43.71	48.12
Serum creatinine (μmol/L)	321	235	208	191	164	170	157
Urinary protein (g/d)	2.980	2.050	0.970	0.770	0.175	0.345	0.378
Plasma albumin (g/L)	34.6	42.0	45.1	44.7	41.5	47.9	45.6

eGFR = estimated glomerular filtration rate.

This case report was prepared in accordance with the Declaration of Helsinki. We have obtained the patient’s written informed consent for the publication of his clinical data and pathological images. According to the policies of Shaanxi Provincial People’s Hospital, for retrospective case reports, no approval from the ethics committee is required when publishing.

## 3. Discussion

In this case report, we described a patient with IgA nephropathy with eGFR < 30 mL/min/1.73 m^2^, who was treated with delayed-release budesonide in combination with cyclophosphamide, after 36 weeks of follow-up, the patient’s urine protein decreased and renal function improved.

The incidence of IgA nephropathy varies by region, ranging from 10% to 40%, and about 40% of patients develop end-stage kidney disease within 20 years.^[9,10]^ The common therapeutic goal for patients with IgA nephropathy is to delay disease progression and prevent further deterioration of renal function by controlling blood pressure and lipids, reducing urinary protein and improving lifestyle.^[11]^ IgA nephropathy is characterized by the deposition of immune complexes containing Gd-IgA1 in the glomerular mesangium, which leads to a series of inflammatory reactions and complement activation, ultimately leading to irreversible glomerulosclerosis, tubular fibrosis, and deterioration of renal function, this Gd-IgA1 is originated from mucosal B cells, which are present in the distal ileum, which is rich in Pyle collection of lymph nodes.^[1,12]^

For patients with eGFR < 30 mL/min/1.73 m², previous guidelines recommended only supportive therapy, however, for high-risk patients with high urine protein and severe renal impairment, supportive therapy alone cannot fundamentally control the progression of the patient’s disease. Studies have shown that glucocorticoid combined with cyclophosphamide significantly reduced hematuria and proteinuria, and improved renal function in patients with IgA nephropathy.^[13–15]^ Delayed-release budesonide, as an oral drug, can be delivered to the distal ileum, where Peyer plaques are highly dense, to reduce the production of Gd-IgA1 and decrease its concentration in the systemic circulation; thus, it can intervene the occurrence and development of IgA nephropathy at the source.^[16]^ Studies have shown that delayed-release budesonide is effective in reducing urinary protein and improving renal function in patients with IgA nephropathy at a high risk of progression.^[17,18]^

Furthermore, studies indicate that the intestinal microbiome plays a crucial role in the development and progression of IgA nephropathy, potentially through its influence on mucosal immune responses and the production of Gd-IgA1.^[19,20]^ Although we did not conduct a detailed dietary assessment or intestinal microbiome analysis for this patient, the use of delayed-release budesonide is intrinsically linked to this concept, as it targets the intestinal lymphoid tissue where pathogenic IgA originates. Our case highlights the potential for treating IgA nephropathy by targeting the intestinal environment. Future studies systematically integrating dietary evaluation and intestinal microbiome analysis will be crucial for further elucidating the specific response of patients to intestinal-targeted therapy.

Most of the current studies have reported IgA nephropathy patients with eGFR > 30 mL/min/1.73 m^2^, and there are few reports on patients with eGFR < 30 mL/min/1.73 m^2^. Studies on delayed-release budesonide combined with cyclophosphamide for IgA nephropathy with an eGFR < 30 mL/min/1.73 m² are extremely limited, Gholizadeh Ghozloujeh Z et al reported a case of a 43-year-old male patient with IgA nephropathy and stage 4 chronic kidney disease who had an eGFR of 28 mL/min/1.73 m^2^, after 12 months of treatment with delayed-release budesonide, his eGFR stabilized and proteinuria decreased.^[21]^ Compared with this case report, our patient had a lower baseline eGFR (20.27 mL/min/1.73 m²), and was treated with a combination therapy of delayed-release budesonide and cyclophosphamide, we observed more significant improvement in renal function. Although there were individual differences, we considered that the addition of cyclophosphamide to delayed-release budesonide may be more beneficial for patients with severely impaired renal function. This result also suggests that even in the advanced stage of the disease, aggressive combination therapy may still improve renal function in patients with intense immune inflammatory activity.

The pathogenesis of IgA nephropathy originates from Gd-IgA1 produced by mucosa-associated B cells, delayed-release budesonide directly reduces the production of pathogenic Gd-IgA1 at its source by inhibiting the activation and differentiation of B cells in the intestinal-associated lymphoid tissue, thereby achieving etiological treatment of the disease. Following deposition of Gd-IgA1 immune complexes in the mesangial region, the complement system is activated, triggering downstream inflammatory responses, leading to acute glomerular damage and rapid decline in renal function. Cyclophosphamide exerts broad immunosuppressive effects by reducing the proliferation and function of both B and T cells. For patients with severely impaired renal function, characterized by coexisting high levels of Gd-IgA1 and glomerular inflammation, using delayed-release budesonide alone may show slower efficacy in controlling rapidly progressing renal dysfunction, while cyclophosphamide alone fails to address the source of the disease. Therefore, combining these 2 drugs aims to rapidly control disease progression.

The KDIGO 2025 Clinical Practice Guideline recommends that patients at risk of progressive loss of renal function with IgA nephropathy should undergo 9 months of delayed-release budesonide treatment.^[22]^ The main difference between our treatment plan and the guideline recommendations lies in the combined use of cyclophosphamide, cyclophosphamide is primarily used in the treatment of rapidly progressive glomerulonephritis, but our patient exhibited a variety of rapid progressing high-risk factors, including severely reduced eGFR, high levels of proteinuria, and chronic sclerotic lesions, considering these factors, our case did not use delayed-release budesonide alone. Instead, it employed delayed-release budesonide as the primary treatment, supplemented with a time-limited course of cyclophosphamide to provide rapid and effective systemic immunosuppression, thereby controlling the disease progression through different mechanisms, and the results showed that delayed-release budesonide combined with cyclophosphamide could reduce the patients’ urinary protein level, and it had a very impressive effect in stabilizing kidney function, and the overall safety was also relatively good. However, this approach should be validated in future prospective studies.

Limitations of this study: First, this is a single-center case report with limited generalizability of its findings, the efficacy and safety of this treatment regimen in a broader population of IgA nephropathy require validation through large-scale prospective randomized controlled trials. Second, although the patient presented with high proteinuria and severe renal impairment before treatment, we cannot completely rule out the possibility of spontaneous clinical improvement. However, considering the chronic nature of the renal pathology and the extremely low eGFR level before treatment, we consider the probability of spontaneous improvement to be extremely low, the observed clinical efficacy is more likely attributable to the active treatment. Finally, this retrospective study with a short follow-up period (36 weeks), we are unable to assess the long-term stability of the kidneys or the risk of relapse, this requires extended observation to determine the long-term efficacy and safety of this treatment regimen. However, for this patient with an extremely low baseline eGFR (20.27 mL/min/1.73 m²), the primary risk lies in rapid progression to end-stage renal disease within the short term, therefore, the improvement in renal function and reduction in proteinuria observed within 36 weeks demonstrate that this combination therapy successfully halted rapid disease progression in the short term. This case provides a valuable clinical idea and preliminary experience for the treatment of advanced high-risk IgA nephropathy with eGFR < 30 mL/min/1.73 m².

## 4. Conclusion

This case demonstrates that for high-risk IgA nephropathy patient with an eGFR < 30 mL/min/1.73 m², in addition to the current KDIGO guidelines’ recommended delayed-release budesonide treatment, a combined short-course cyclophosphamide treatment regimen can effectively improve the patient’s renal function and reduce proteinuria. This approach aligns with guideline recommendations for targeted therapy while supplementing treatment for extreme populations inadequately covered by guidelines. However, its long-term efficacy and safety require further validation through large-sample, prospective studies.

## Acknowledgments

The authors sincerely appreciate the time and effort of all who contributed.

## Author contributions

**Conceptualization:** Zhuan'e Yao, Peng Zhang.

**Data curation:** Pengbo Wang.

**Methodology:** Zhuan'e Yao, Pengbo Wang, Qinjuan Fu, Peng Zhang.

**Software:** Zhuan'e Yao, Pengbo Wang, Qinjuan Fu, Peng Zhang.

**Writing – original draft:** Zhuan'e Yao.

**Writing – review & editing:** Peng Zhang.
